# Corneal hysteresis in patients with glaucoma-like optic discs, ocular hypertension and glaucoma

**DOI:** 10.1186/s12886-016-0396-9

**Published:** 2017-01-10

**Authors:** Melissa L. Murphy, Olya Pokrovskaya, Marie Galligan, Colm O’Brien

**Affiliations:** School of Medicine and Medical Sciences, Mater Misericordiae University Hospital, Eccles Street Dublin 7, Dublin, Ireland

**Keywords:** Corneal hysteresis, Corneal resistance factor, Glaucoma-like optic discs, Central corneal thickness, Ocular response analyser

## Abstract

**Background:**

To compare corneal hysteresis (CH) measurements between patients with glaucoma, ocular hypertension (OHT) and glaucoma-like optic discs (GLD)- defined as a cup to disc ratio greater than or equal to 0.6 with normal intraocular pressure (IOP) and visual fields. The secondary aim was to investigate whether corneal resistance factor (CRF) and central corneal thickness (CCT) differ between patient groups.

**Methods:**

In this cross sectional study a total of 123 patients (one eye each) were recruited from a glaucoma outpatient department to undergo ocular response analyser (ORA) testing and ultrasound pachymetry as well as clinical examination. A One-way Analysis of Covariance (ANCOVA) was conducted to evaluate the mean difference in CH between the three diagnostic groups (glaucoma, OHT and GLD) correcting for potential confounding factors, IOP and age. Analysis was repeated for CRF and CCT.

**Results:**

There was a significant difference in mean CH across the three diagnosis groups; F(2, 115) = 96.95; *p* < 0.001. Mean CH significantly higher for GLD compared to glaucoma (mean difference 1.83, *p* < 0.001), and significantly higher for OHT compared to glaucoma (mean difference 2.35, *p* < 0.001). Mean CH was slightly lower in patients with GLD than those with OHT but this difference was not statistically significant. A similar pattern was seen when the analysis was repeated for CRF and CCT.

**Conclusions:**

Higher CH in GLD and OHT compared to glaucoma suggests increased viscoelasticity of ocular tissues may have a protective role against glaucoma.

## Background

Glaucoma is a leading cause of irreversible blindness worldwide. A recent meta-analysis estimated a significant increase in the incidence of glaucoma over the next three decades worldwide – from 64.3 million affected in 2013, rising to 76 million in 2020, and to 111.8 million in 2040 [1]. Glaucoma is defined as a multifactorial optic neuropathy characterised by the accelerated loss of retinal ganglion cells resulting in peripheral visual field loss. Age and raised intraocular pressure (IOP) are considered the main risk factors for glaucoma. Reduction in IOP, either by medical or surgical intervention, is currently the only proven treatment option [2]. It is estimated that 30–50% of glaucoma patients have normal IOP [3, 4] suggesting that other factors, including tissue biomechanics, may have a role in glaucoma risk and optic nerve damage progression.

Recent studies have investigated the relationship between corneal structural properties and glaucoma. Corneal biomechanics offer us an insight into how the cornea behaves in certain situations and may thus be used to extrapolate optic nerve susceptibility to certain stressors. The physical composition of the cornea gives it viscoelastic properties, meaning it demonstrates elements of both viscosity and elasticity. Corneal hysteresis (CH) is defined as the viscous dampening of the cornea and reflects the ability of the cornea to absorb and dissipate energy [5, 6]. CH has a potential role as an IOP correction factor and as a proxy marker of an individual’s susceptibility to glaucomatous optic neuropathy [5]. In addition, corneal resistance factor (CRF) reflects the elastic properties of the cornea i.e. its ability to deform reversibly under stress and appears to be an indicator of corneal resistance. Both can be measured using the Ocular Response Analyser (ORA) (Reichert, Corp.; NY, USA) which involves applying an air pulse of a defined magnitude to the surface of the cornea and measuring the difference between the two applantation measurements during corneal deformity and return to its original configuration [6].

The principal aim of our study was to determine if CH differs between patients with glaucoma, ocular hypertension (OHT) and glaucoma-like optic discs (GLD). The secondary aim was to investigate whether CRF and central corneal thickness (CCT) differ between these patient groups. We had a particular interest in GLD. To our knowledge, there have been no studies to date looking at the relationship between CH and GLD.

## Methods

### Subjects

In this cross-sectional single-centre observational study a total of 123 patients were recruited at the glaucoma clinic in the Mater Misericordiae Hospital, Dublin, Ireland between July 2014 and July 2015. Both eyes from each patient were analysed using the ORA and one eye was chosen by random selection from each patient generating 123 eyes for inclusion in the study. Informed consent was obtained from all individual participants included in the study. Patients with a diagnosis of high tension glaucoma (HTG) (*n* = 37), pseudoexfoliative glaucoma (PXFG) (*n* = 12), OHT (*n* = 28), NTG (*n* = 24) and GLD (*n* = 22) were recruited. The diagnosis was determined by a glaucoma specialist at a previous visit. Exclusion criteria were unreliable visual fields or previous glaucoma surgeries. The protocol of the study adhered to the tenets of the Declaration of Helsinki.

### Examination techniques

Diagnosis, IOP, CCT, CH and CRF was recorded for each patient. POAG referred to high or low pressure open angle glaucoma of unknown aetiology. High tension open angle glaucoma (HTG) was defined as a raised IOP of greater than 21 mmHg measured using Goldmann applanation tonometry (GAT), glaucomatous optic disc changes, an open drainage angle, and characteristic visual field defects on a Humphrey perimeter central 24-2 threshold test. Normal tension open angle glaucoma (NTG) was defined as glaucomatous disc changes and visual field loss without an IOP measurement exceeding 21 mmHg at any clinic visit. PXFG was defined as exfoliation material visualised within the anterior segment of the eye resulting in secondary open angle glaucoma. OHT was diagnosed on the basis of repeated IOP measurements of over 21 mmHg without evidence of glaucomatous nerve damage or visual field loss. For the purpose of this study GLD was defined similarly to the Tomita et al. study as an increased cup-to-disc ratio (≥0.6) and pallor, asymmetry of cupping between eyes in a patient with normal IOP, normal visual fields, and open angles [7].

The primary aim of this study was to determine whether CH differs between patients with glaucoma, OHT, and GLD. Therefore, CH is the primary outcome measure in this study. The secondary aim was to investigate whether CRF and CCT differ between these patient groups. For the purposes of statistical analysis NTG, HTG and PXFG patients were grouped together as “glaucoma”. Each of these subgroups demonstrate evidence of a glaucomatous optic neuropathy with corresponding visual field changes.

The CH and CRF were measured using the Reichert ORA. Four measurements were taken for each selected eye and the average value was used in the analysis. CCT was measured in the same eye using a Pachmate 55 handheld ultrasonic pachymeter (DGH, Exton.; PA, USA) after instillation of one drop of topical proxymethacaine 0.5%.

### Statistical analysis

Data including patient age, gender, diagnosis, CH, CRF and CCT was collated using Microsoft Excel and analysed using the statistical package IBM SPSS (version 20). Clinical information on patients was summarised by diagnosis group. Normality of each outcome variable was assessed using histograms and Shapiro-Wilk normality tests. One-way Analysis of Covariance (ANCOVA) was conducted to test for a difference in mean CH between the three diagnosis groups (glaucoma, OHT and GLD), whilst controlling for possible confounding factors – IOP and age. IOP and age are both potential confounding factors for CH analysis, as lower CH is associated with higher IOP and older age, and these were corrected for in our analysis.

All assumptions of ANCOVA including homogeneity of variance, homogeneity of regression slopes and homoscedasticity were tested before analysis. Bonferroni post hoc testing was used for conducting pairwise comparisons of CH between diagnosis groups. Similar analysis was carried out for CRF and CCT. Scatter plots and Pearson correlation coefficients were used to assess the relationships between CH and age, and between CH and CCT. In this study, a *p* value < 0.05 was considered statistically significant.

## Results

### Subjects

The study included one eye from each of 123 patients. Table 1 summarises the basic clinical information for the patients evaluated in this study in the three different diagnosis groups. There is a higher percentage of females (67.9%) in the GLD group compared to the OHT (53.4%) and glaucoma (45.5%) groups, and the GLD patients were a little younger on average than patients in the other two groups. Mean Goldmann-corrected IOP measured with ORA was higher in the GLD group (19.9 mmHg) than in the OHT and glaucoma groups (17.4 and 17.6 mmHg respectively).

**Table 1:** Demographics and characteristics of the three diagnosis groups included in the study (Glaucoma, GLD and OHT)

	Glaucoma (*n* = 73)	GLD (*n* = 22)	OHT (*n* = 28)	Total (*n* = 123)
Female	10 (45.5%)	39 (53.4%)	19 (67.9%)	68 (55.3%)
Male	12 (54.5%)	34 (46.6%)	9 (32.1%)	55 (44.7%)
Age (years)	67 ± 14	70 ± 12	62 ± 14	67 ± 13
IOPg (mm Hg)	17.6 ± 4.5	17.4 ± 4.1	19.9 ± 4.0	18.0 ± 4.3
CH (mm Hg)	9.9 ± 1.7	8.0 ± 2.0	10.3 ± 1.5	8.8 ± 2.1

Gender is summarised by frequency and percentage; other variables by mean ± SD

*GLD* glaucoma-like optic discs, *OHT* ocular hypertension, *IOPg* Goldmann-corrected IOP, *CH* corneal hysteresis, *SD* standard deviation

### Differences in CH, CRF and CCT between patient subgroups

**Fig. 1:**
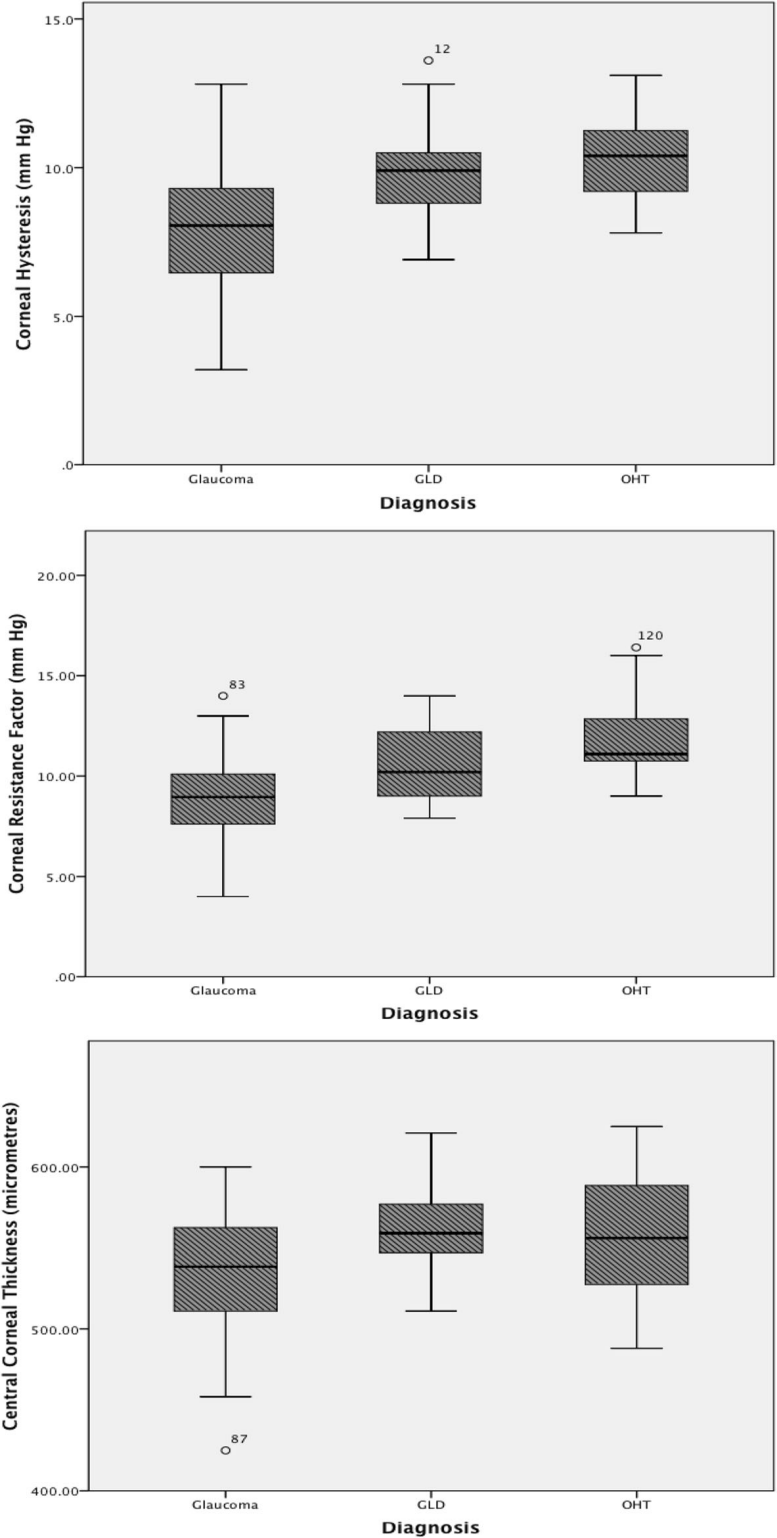
Differences in CH, CRF and CCT between patient subgroups. *Box and whiskers* plots illustrating the difference in mean and interquartile range of corneal hysteresis (CH) (*Top*), corneal resistance factor (CRF) (*Middle*), and central corneal thickness (CCT) (*Bottom*) between the three diagnosis subgroups (glaucoma-like discs [GLD], glaucoma, ocular hypertension [OHT]). CH and CRF is higher in both OHT and GLD compared to glaucoma. A similar pattern was demonstrated in CCT across patient subgroups

Boxplots were used to compare the distribution of CH (Fig. 1, Top), CRF (Fig. 1, Middle) and CCT (Fig. 1, Bottom) across the three diagnostic groups. Table 2 shows the results of ANCOVA with CH as the dependent variable. It reveals a significant difference in mean CH across the three diagnosis groups; F(2, 115) = 96.95; *p* < 0.001. Age and IOP were controlled for in this analysis but were not found to be significant.

**Table 2:** ANCOVA showing significantly different CH in diagnosis groups correcting for IOP and age

Test of between-subject effects						
Source	Type III sum of squares	df	Mean square	F	Sig.	Partial Eta Squared
Corrected Model	146.422^a^	4	36.605	10.55	.000	.268
Intercept	336.386	1	336.386	0	.000	.457
Diagnosis	117.977	2	58.989	96.95	.000	.228
Age	4.105	1	4.105	3	.279	.010
IOPg	10.916	1	10.916	17.00	.079	.027
Error	399.002	115	3.470	2		
Total	9968.920	120		1.183		
Corrected Total	545.424	119		3.146		

*ANCOVA* one-way analysis of covariance, *CH* corneal hysteresis, *IOPg* Goldmann-correlated IOP, *df* degrees of freedom, *Sig.* significance level

^a^R Squared = .268 (Adjusted R Squared = .243)

Post hoc pairwise comparisons of CH between diagnosis groups were conducted, using the Bonferroni method to correct for multiple testing error (Table 3). Mean CH was found to be significantly higher for GLD compared to glaucoma (mean difference 1.83, *p* < 0.001). Mean CH was also found to be significantly higher for OHT compared to glaucoma (mean difference 2.35, *p* < 0.001). Mean CH was slightly lower in patients with GLD than those with OHT but this difference was not statistically significant.

**Table 3:** Bonferroni post-hoc analysis of CH across diagnosis groups

Comparison	Mean difference	Std. error	Sig.^b^	95% confidence interval for difference^b^	
				Lower bound	Upper bound
GLD vs Glaucoma	1.832^a^	0.46	0	0.715	2.95
OHT vs GLD	0.516	0.546	1	−0.81	1.841
OHT vs Glaucoma	2.348^a^	0.451	0	1.253	3.443

Based on estimated marginal means

*CH* corneal hysteresis, *GLD* glaucoma-like discs, *OHT* ocular hypertension, *Sig.* significance level

^a^The mean difference is significant at the

^b^Adjustment for multiple comparisons: Bonferroni

These results are also reflected in the boxplots of CH by diagnosis group (Fig. 1a), showing lower CH values in general for patients with glaucoma, compared to the OHT and GLD diagnosis groups. A similar pattern was seen when the analysis was repeated for CRF and CCT. Mean CRF was significantly higher in GLD compared to glaucoma (mean difference 1.47, *p* < 0.01), and significantly higher in OHT compared to glaucoma (mean difference 2.17, *p* < 0.01). We also demonstrated that CCT is significantly thicker in GLD compared to glaucoma (mean difference 27.5 μm, *p* < 0.01) and CCT is also significantly thicker in OHT compared to glaucoma (mean difference 20.0 μm, *p* = 0.03).

### Correlations between CH and CCT and patient age

**Fig. 2:**
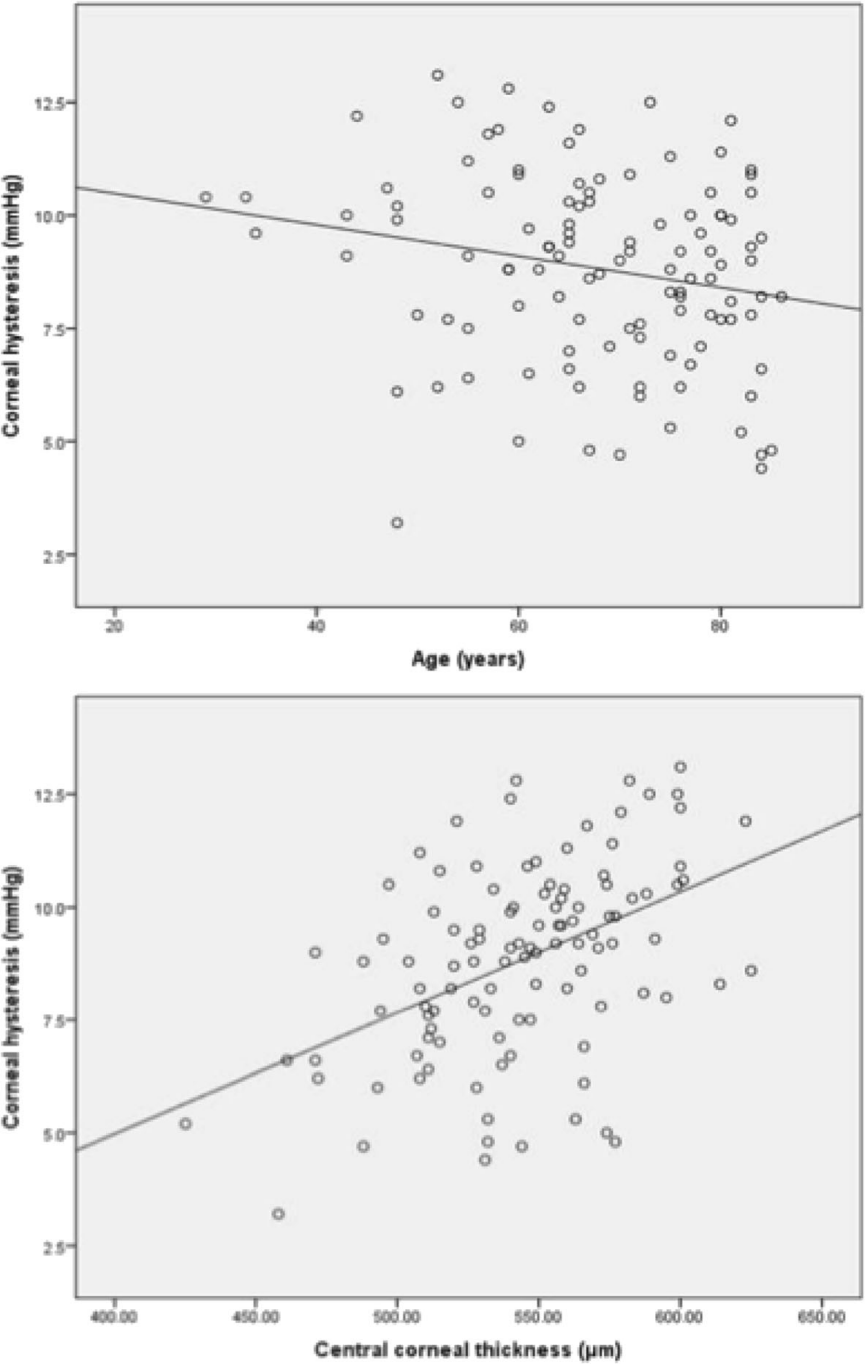
Correlations between CH and age and also between CH and CCT. The relationships between corneal hysteresis (CH) and age (*Top*), and CH and central corneal thickness (CCT) (*Bottom*). There is a weak negative correlation between CH and age (*r* = −0.22, *p* = 0.01) and a strong positive correlation between CH and CCT (*r* = 0.40, *p* = <0.001)

Finally, the scatter plots (Fig. 2) illustrate the relationship between CH and age, and the relationship between CH and CCT across all groups pooled together. These plots demonstrate a negative correlation between CH and age (*r* = −0.22, *p* = 0.005) and a strong positive correlation between CH and CCT (*r* = 0.40, *p* = <0.001).

## Discussion and conclusions

This work provides novel insight into the interplay of corneal biomechanical properties and glaucoma. Our results are consistent with previous research in demonstrating higher CH in patients with a diagnosis of OHT as compared to patients with glaucoma [8]. In a prospective observational study Shah et al. compared CH and CRF between patients with OHT, NTG and POAG and found CH to be highest in the OHT group in keeping with our findings [8]. This suggests that higher CH may have a protective role in patients with raised IOP. Furthermore, our study found a significantly higher CH in patients with GLD compared to glaucoma patients. A similar pattern was also seen in the CRF values between the groups, suggesting greater viscoelasticity of ocular tissue in GLD and OHT patients than in glaucoma patients. This adds to the body of evidence surrounding the diagnostic promise of corneal hysteresis, and the hypothesis that increased viscoelasticity of ocular tissues may be protective against glaucomatous nerve damage.

Our work also demonstrated that CCT was significantly thicker in both the OHT and GLD groups compared to glaucoma patients. This may reflect an inherent resilience against glaucomatous tissue damage in the GLD and OHT subgroup. CCT has been shown to be a protective factor in patients against glaucoma in numerous previous studies [9–11]. We demonstrated a weak negative correlation between CH and age, as well as a strong positive correlation between both CH and CRF with CCT. This would suggest thicker corneas may be more compliant and reflect a reduced susceptibility to high IOP within the eye, as well as the established fact that thicker corneas may result in falsely elevated IOP measurements.

A number of studies have been published in the area of corneal biomechanics in glaucoma patients. These findings are strongly suggestive that corneal viscoelasticity is altered in the disease progress. Kotecha et al. described an IOP-independent corneal factor (corneal constant factor; mm Hg) and demonstrated that this parameter increased with thicker CCT and decreased with age [12]. Other studies have also found an association between greater CH, greater CCT, and lower IOP [6, 13, 14]. One such study looking at 207 normal eyes demonstrated only a moderate correlation between CH and increasing CCT suggesting CH measurements may reflect different aspects of biomechanical rigidity [6]. In contrast, a study on patients with unilateral POAG found that CH but not CRF was significantly correlated with IOP concluding that once CH is corrected for IOP, there is no difference in corneal biomechanical properties between eyes with and without POAG [13]. Due to these findings it is important to take both CH and CRF into consideration. Interestingly, a number of recent publications suggest faster rates of glaucomatous progression in patients with a lower CH. This is evidenced by accelerated progression of visual field defects [9, 15, 16].

Regarding GLD, a study conducted by Tomita et al. in 1989 first described this subgroup as a variant of glaucoma with increased cupping and pallor, superior or inferior extension of cupping with pallor and asymmetry of cupping, and pallor between eyes without associated increased IOP or visual field loss [7]. The study compared 48 GLD patients to 48 primary open angle glaucoma (POAG) patients in relation to optic disc fluoroscein angiography, family history of glaucoma and retinal nerve fiber layer defects with no statistically significant difference between groups concluding that GLD may be a variant of POAG. Furthermore, another study of GLD patients comparing glaucoma-like discs to optic discs in normal controls using stereophotogrammetry revealed a significant reduction in retinal nerve fiber layer thickness in GLD compared to healthy controls, again suggesting GLD may be a variant of glaucoma [17].

The results of our study support the growing body of evidence for the importance of the biomechanical properties of ocular tissues in the pathophysiological glaucomatous process. This means structural changes, such as altered tissue compliance, especially at the optic nerve, may reflect altered susceptibility in individual patients to both disease emergence and progression. This may have particular significance in patients who have disease despite normal IOP (i.e. NTG) and in the required follow-up of patients with suspect discs who are yet to demonstrate visual field loss. It is also known that CH and CRF are affected by age such that age-associated tissue remodelling is reflected in a reduction of CH. This has been demonstrated in studies on corneal biomechanics in older healthy volunteers compared to younger counterparts in which a significantly reduced CH was seen with ageing [18, 19].

Ageing is associated with reduced elasticity and therefore reduced compliance in tissues throughout the body [20–23]. This process is believed to be a combination of both oxidative stress caused by reactive oxygen species and the formation of advanced glycosylation end products caused by the non-enzymatic glycation of proteins [24]. These, in combination, result in haphazard cross-linking of proteins, altering tissue architecture and resulting in reduced elasticity. A reduction in mechanical compliance with age has been demonstrated in the cornea [22], the lamina cribrosa [20], the sclera [21], the ciliary muscle, and lens [23]. In 2014, the EPIC-Norfolk Eye Study found CH to be negatively correlated with both linear cup-to-disc ratio and increasing age [18]. In addition, hormonal factors may impact on the biomechanical properties of the cornea, with oestrogen believed to have a protective role [25, 26]. Due to the substantial evidence that ocular biomechanical response to increased IOP affects the degree of glaucoma damage this is becoming an exciting new target for the development of glaucoma therapies [27].

The significance of our results are that CH may provide further information on the reason why some individuals with a high cup:disc ratio and normal IOP develop NTG whereas others do not. To our knowledge we are the first group to look at CH in patients with GLD compared to patients with demonstrable glaucoma and we find it to be significantly higher in this patient cohort. IOP measurements as well as CCT, optic disc characteristics and visual field defects are routinely reported in patients at risk of this disease. However, altered tissue biomechanics may have an important role to play in both the diagnosis and the monitoring of disease progression, and are not adequately accounted for in the aforementioned parameters. The inclusion of CH and CRF measurements as part of a routine follow-up examination or even as part of an initial assessment may help to individualise patient therapy resulting in improved and tailored management. A direct measurement of optic head tissue compliance may be more relevant in glaucoma patients. A recent paper by Li et al. in 2016 suggested lamina cribrosa depth analysis using enhanced depth imaging optical coherence tomography can help differentiate HTG from normal eyes as it is more posteriorly located in HTG [28].

Limitations of our study include that patients were not followed up over a period of time to assess CH as an independent risk factor in the emergence of disease in glaucoma suspects and OHT patients, as well as a potential risk factor in the disease progression of known glaucoma patients. Also HTG, PXFG and NTG were grouped together as glaucoma patients for the purpose of analysis whereas separate analysis of each of these groups could yield more accurate results.

Future research in the area of CH should focus on its role in other diseases characterised by altered tissue compliance such as diabetes and hypertension. These areas may reflect a further advantage for the addition of CH measurements into routine ophthalmological examinations.

## Abbreviations

ANCOVA: One-way analysis of covariance
CCT: Central corneal thickness
CH: Corneal hysteresis
CRF: Corneal resistance factor
GLD: Glaucoma-like optic discs
HTG: High tension glaucoma
IOP: Intraocular pressure
NTG: Normal tension glaucoma
OHT: Ocular hypertension
ORA: Ocular response analyser
POAG: Primary open angle glaucoma
PXFG: Pseudoexfoliation glaucoma
